# Differences between Novice and Expert Raters Assessing Trunk Control Using the Trunk Control Measurement Scale Spanish Version (TCMS-S) in Children with Cerebral Palsy

**DOI:** 10.3390/jcm12103568

**Published:** 2023-05-19

**Authors:** Javier López-Ruiz, Cecilia Estrada-Barranco, Maria José Giménez-Mestre, Isabel Villarroya-Mateos, Patricia Martín-Casas, Ibai López-de-Uralde-Villanueva

**Affiliations:** 1 Department of Physiotherapy, Faculty of Sport Sciences, Universidad Europea de Madrid, Villaviciosa de Odón, 28670 Madrid, Spain; javier.lopez3@universidadeuropea.es (J.L.-R.); cecilia.estrada@universidadeuropea.es (C.E.-B.); mariajose.gimenez@universidadeuropea.es (M.J.G.-M.); 2 Doctoral Program in Healthcare, Faculty of Nursing, Physiotherapy and Podiatry, University Complutense of Madrid, 28040 Madrid, Spain; 3 Department of Radiology, Rehabilitation and Physiotherapy, Faculty of Nursing, Physiotherapy and Podiatry, Universidad Complutense de Madrid, 28040 Madrid, Spain; ibailope@ucm.es; 4 Faculty of Health Sciences, Universidad San Jorge, Villanueva de Gállego, 50830 Zaragoza, Spain; ivillarroya@usj.es; 5 InPhysio Research Group, Instituto de Investigación Sanitaria del Hospital Clínico San Carlos (IdISSC), 28040 Madrid, Spain

**Keywords:** cerebral palsy, trunk control, postural control, assessment, inter-rater reliability, novice-expert, psychometric properties

## Abstract

The Trunk Control Measurement Scale (TCMS) is a valid and reliable tool to assess static and dynamic trunk control in cerebral palsy. However, there is no evidence informing about differences between novice and expert raters. A cross-sectional study was conducted with participants between the ages of 6 and 18 years with a CP diagnosis. The TCMS Spanish version (TCMS-S) was administered in-person by an expert rater, and video recordings were taken for later scoring by the expert and three other raters with varying levels of clinical experience. The intraclass correlation coefficient (ICC) was used to evaluate reliability between raters for the total and subscales of the TCMS-S scores. Standard Error of Measurement (SEM) and Minimal Detectable Change (MDC) were also calculated. There was a high level of agreement between expert raters (ICC ≥ 0.93), while novice raters demonstrated good agreement (ICC > 0.72). Additionally, it was observed that novice raters had a slightly higher SEM and MDC than expert raters. The Selective Movement Control subscale exhibited slightly higher SEM and MDC values compared to the TCMS-S total and other subscales, irrespective of the rater’s level of expertise. Overall, the study showed that the TCMS-S is a reliable tool for evaluating trunk control in the Spanish pediatric population with cerebral palsy, regardless of the rater’s experience level.

## 1. Introduction

Cerebral palsy (CP) is defined as a group of disorders in the development of movement and posture, attributed to non-progressive brain lesions that occur during the perinatal stage, leading to limitations in activities and participation [1,2]. Children and youth with CP present a great heterogeneity of clinical forms and severity levels that are challenging to assess and measure [3,4]. It is essential to have reliable and valid assessment tools to identify weaknesses as well as strengths for each International Classification of Functioning (ICF) dimension in this population [5,6,7].

Trunk control has been shown to be a key function in the development of postural and movement control [8,9], seems to be related to the level of performance in the activities [10,11], and is also related to independence in self-care and mobility [12]. Wallard et al. [13,14] and Pierret et al. [15,16] investigated the importance of head and trunk control in children with CP. They found that children with CP have difficulty stabilizing their head and controlling their trunk during movement, which can affect their ability to walk and perform daily activities independently. Recent studies also found that a rehabilitation program focused on trunk postural activities can improve trunk control and walking ability in children with CP [16,17]. These findings highlight the importance of evaluating and addressing trunk control in the rehabilitation of children with CP. 

The Trunk Control Measurement Scale (TCMS) is an assessment tool that measures static and dynamic trunk control, providing qualitative information on functioning in the three planes of space: frontal plane (inclinations), sagittal plan (flexion-extension), and transversal plane (rotations) [18,19]. It consists of three sub-scales: static sitting balance, selective motor control, and dynamic reaching. Various cultural adaptations of the TCMS have been developed, and each of them has demonstrated comparable psychometric properties to the original version. This confirms their suitability for both clinical and research purposes [18,19,20,21,22,23,24]. TCMS Spanish version (TCMS-S) has recently been adapted to Spanish children and youth with CP demonstrating adequate psychometric properties similar to those of the original TCMS scale [25].

Because CP is a lifelong condition, it is common for patients to be assessed by different raters over time. The results of assessments can be influenced by the level of experience and specific training of the rater, leading to differences from scores obtained by expert raters. There is evidence for this issue for widely used scales as the GMFM [26], the Test of Gross Motor Development [27,28], and the Balance Error Scoring System (BESS) [29], where novice raters have shown lower agreement and, consequently, higher Standard Error of Measurement (SEM) and Minimal Detectable Change (MDC). It is important to note that higher SEM and MDC indicate lower accuracy when interpreting results of clinical studies, assessing a patient’s functional capacity, or making clinical decisions. However, while there have been several studies exploring the reliability of the TCMS [12,18,20,21,22,25], none have previously explored the reliability of rater type, i.e., novice vs. expert. It seems important to verify that the TCMS-S maintain its psychometric properties independently of the rater and his/her experience [30]. 

The aim of the present study is to analyze the differences between ratings performed with the TCMS-S by expert and novice raters. 

## 2. Materials and Methods

### 2.1. Study Design, Setting and Participants

The study was conducted using a cross-sectional design that followed the guidelines set by the Consensus-based Standards for the Selection of Health Status Measurement Instruments (COSMIN) [31]. The study received approval from the ethics committee of the Bio-Medical Foundation of the Hospital Infantil Universitario Niño Jesus of Madrid (registration number: R-0066/20) and was carried out in compliance with the Declaration of Helsinki.

Participants were recruited from the schedules of the traumatology and rehabilitation services at Hospital Infantil Universitario Niño Jesus in Madrid, using a method of consecutive sampling by convenience. Inclusion criteria were diagnostic of CP, age between 6 and 18 years, able to sit without trunk or feet support and to follow test instructions, and Gross Motor Function Classification System (GMFCS) levels I-IV. Exclusion criteria were orthopedical surgery or botulinum toxin injection during the last 6 months [12]. Before initiating any action related to the study, informed consent was obtained from the families of all participants. Additionally, participants over 12 years old provided written consent, while those under 12 years old provided verbal consent to participate in the study.

### 2.2. Trunk Control Measurement Scale-S

The TCMS-S scale evaluates seated trunk control across three dimensions, with a maximum score of 58 points. These dimensions are static balance (20 points), selective movement control (28 points), and dynamic reaching (10 points). Each item is scored on a scale of 0 to 3, where 0 indicates an inability to perform the task and 3 represents complete performance. The test is active, and the evaluator provides verbal instructions, demonstrates movements visually, or guides the participant before asking them to perform the test. The best score from three attempts is recorded, and administration typically takes between 15 and 20 min [18]. 

### 2.3. Study Procedures

First, all raters were previously instructed about the evaluation through TCMS-S by the gold standard expert. A two-hour training session was held for all raters, during which the TCMS-S scale was presented, training videos were analyzed, and the score was practiced. Any doubts that may have arisen were also answered [29]. The gold standard expert was a physical therapist with over ten years of clinical experience in the specific field of pediatric care, as well as five years of experience using the TCMS scale. The other expert rater was a physical therapist with over ten years of clinical experience in pediatric care but without any experience using the TCMS scale. The novice raters consisted of a master’s student in physical therapy and a physical therapist without any clinical experience in pediatrics. Neither of the novice raters had any prior experience with the TCMS scale [18,19,26,29]. The gold standard expert administered the TCMS-S scale to the participants with CP in a face-to-face clinical setting. These assessments were also video recorded for later scoring by all raters [12,27,29]. The video-assessment was performed by this researcher and the three other raters with different levels of clinical experience and experience with the scale. During video scoring, raters were allowed to pause the video recordings and to review items as many times as necessary [26]. All raters were blinded to others during the study period [27,29].

All requisites for protection of personal data were met. The processing, communication, and transfer of personal data of all patients complied with the provisions of Organic Law 03/2018 of 5 December on the Protection of Personal Data and the Guarantee of Digital Rights as well as Regulation (EU) 2016/679 of the European Parliament and of the Council of 27 April 2016 on Data Protection (GDPR).

All data were recorded on an evaluation sheet, checked before the end of the assessment, and clarified any participant’s or parent’s doubts [18,19]. 

### 2.4. Data Analysis

The data were analyzed using SPSS v. 29 software (SPSS Inc., Chicago, IL, USA). The level of significance was set at *p* < 0.05. A normal distribution of the variables was assumed based on the results of the assumption tests, as well as on the central limit theorem (due to the large sample size; N > 30).

The intraclass correlation coefficient (ICC) by a 2-way fixed-effect model was used to evaluate the inter-rater reliability for the absolute value of the total and subscales TCMS scores. ICC values were interpreted as follows: excellent (ICC ≥ 0.90); good (0.90 > ICC ≥ 0.70); fair (0.70 > ICC ≥ 0.40); and poor (ICC < 0.40) [32]. The precision of the reliability results was measured by the standard error of measurement (SEM), which was calculated as standard deviation of the difference score/$\sqrt{2}$. In addition, the minimal detectable change, required to be 95% confident that the observed change between 2 measurements reflects true change and not measurement error, was calculated (MDC95 = $SEM\times \sqrt{2}\times 1.96$) [33].

## 3. Results

A total of 96 participants were included in the present study, of whom 44 were female, with a mean age of 12.5 ± 3.3 years (range 6–18 years). Among them, 47 participants were between 6 and 12 years old with a mean age of 9.6 ± 2.2, and 49 participants were between 12 and 18 years old with a mean age of 15.1 ± 1.8 years. Table 1 presents the distribution of participants by diagnoses and functional levels according to GMFCS for each age group.

The means and standard deviations of the total scores obtained by novices and experts, as well as the TCMS-S subscales, were calculated and grouped based on the GMFCS level. These data are presented in Table 2. Higher total TCMS-S scores and subscale scores are observed in participants with higher functional GMFCS levels, regardless of the rater’s experience level.

The scoring of the video-recorded assessments was conducted by four raters: two experts and two novices. Both expert raters were physical therapists with specific postgraduate training in pediatric physical therapy. One of the experts (ER1) had 20 years of clinical experience in pediatric physical therapy, as well as experience using the TCMS-S, and was consider the gold standard expert (ER1) [34]. The other expert (ER2) had 15 years of clinical experience in pediatric physical therapy but initially was not familiar with the TCMS-S. The novice raters (N1 and N2) were physical therapists without prior experience in pediatric physical therapy or TCMS-S. N1 had graduated 10 years ago, while N2 was a recent graduate. The mean age of raters was 35.8 years (range 25–42 years), and 75% of them (NR1, NR2, and ER2) were women. 

Table 3 summarizes data regarding the inter-rater reliability for the total TCMS-S score and subscales. Regardless of whether inter-rater reliability was assessed for the total scale or for the sub-scales, the agreement between experience raters was excellent (ICC ≥ 0.93). Novice raters had slightly lower agreement than experienced raters, with good inter-rater reliability for the total TCMS-S score and subscales (ICC = 0.72–0.82). Thus, novice raters obtained higher SEM and MDC than experienced raters. However, irrespective of the rater’s degree of experience, the highest SEM was found in the “Selective movement control” subscale, followed by “Static sitting balance” and “Dynamic reaching”.

Regarding agreement between experienced and novice raters, excellent reliability was observed for the TCMS-S total score and its subscales (ICC ≥ 0.93), except for the “Selective movement control” subscale, where the observed reliability was rated as good (ICC = 0.85). The SEM and MDC established for agreement between expert and novice raters on the TCMS-S total score were 3.1 points and 8.7 points, respectively. The highest degree of measurement error was found in the “Selective movement control” subscale (SEM = 2.3, MDC = 6.3).

## 4. Discussion

The aim of this study was to explore the expertise influence on assessing trunk control in children with CP using TCMS-S. The results indicated that regardless of the level of experience, the agreement between raters was good to excellent for the total TCMS-S score and its subscales.

The distribution of participants according to GMFCS levels showed that most participants with unilateral impairment had level I (63.9%), while those with diparesis were distributed between levels I (36.3%) and II (54.5%). This distribution coincided with that reported in previous studies in which children with hemiparesis presented higher functional levels, and children with bilateral involvement unfilled all GMFCS levels [3,35].

Participants with better motor abilities and walking skills showed greater trunk control. These findings are consistent with previous studies, including Heyrman et al. (2013) and Balzer et al. (2018) who respectively analyzed original and German versions of the TCMS scale. Their results showed that children with greater trunk limitation exhibit lower walking ability and functional motor skills related with lower limb [36,37]. Additionally, we examined the score differences between expert and novice raters and found that experts assigned slightly higher scores in GMFCS levels I and II. However, these differences were not observed in GMFCS levels III and IV, and the trend was inverted for some subscales. Therefore, no definitive trend was observed. 

Among the raters, although only ER1 had previous experience in the use of TCMS-S, a comparison was conducted between two expert raters (ER1 and ER2) with more than ten years of experience in pediatric physiotherapy and novice raters (N1 and N2) without experience in the field of pediatric physiotherapy nor in the use of the scale. Such comparisons are common in similar studies [26,27,29,38]. However, to our knowledge, while intra- and inter-rater differences have been analyzed for TCMS, there has been no research on differences among raters with varying levels of experience [12,18,19,20,21,22,24].

With respect to the procedure for the evaluation, in the present study, TCMS-S was scored by all raters using video recordings. TCMS-S considers compensations and lower amplitude of movements to evaluate performance quality [18,36], so raters need to detect differences in the execution of the different items measured by TCMS-S to ensure consistent scoring. The use of video recordings during the assessment provides increased safety for the patient [26], as well as greater objectivity and reduced potential for facilitation or bias based on test results [38,39]. 

The TCMS-S total and subscales demonstrated excellent inter-rater reliability among expert raters (ICC ≥ 0.93). Similar results were observed when comparing the ratings of experts and novice raters, except for the Selective Movement Control subscale (ICC = 0.85). These findings are consistent with previous studies that have examined the reliability of different versions of the TCMS [12,18,20,22,40]. However, there were differences in the procedures of all these studies: Heyrman et al. [18], in the original version, compared scores of two trained raters who scored live but independently. Marsico et al. [12], in the German version, compared the scores of two expert raters, with more than 10 years of clinical experience, who administered the scale in real-time but who scored from the recorded videos. Heo et al., in the Korean version [24], compared results of four raters with more than 8 years of experience in pediatric physical therapy. While they reported that these four raters scored the videos, it remains unclear who administered the scale. The remaining versions [20,21,22] indicate that two raters scored the assessments, but it remains unclear whether they evaluated at two different times or during the same session, as well as their experience level. As a whole, findings suggest that all versions of the TCMS have excellent inter-rater reliability, even when comparing live versus video assessments [12,18]. However, this statement should be approached with caution due to the differences between the procedures and the lack of information on raters’ experience and scoring modes. Further research comparing TCMS scores between live and video ratings could shed light on this aspect.

In the present study, while novice raters had slightly lower agreement than experienced raters, inter-rater reliability was still good for the total TCMS-S score and subscales (ICC ≥ 0.72). In line of these findings, Kuo et al. [29] found excellent reliability when comparing ratings of videorecorded assessments of the BESS scale by expert raters in pediatric physical therapy who were familiar with the BESS scale. In addition, they also found good reliability among novice raters regardless of whether they had received specific training or not. These results could be related with previous training about the scale, consisting of an online module with 4 examples of BESS scale evaluation, which included written instructions and required a 100% match in the scoring of the proposed examples [29]. In the present study, participants received two-hour-long training sessions from the gold standard expert (ER1) in online education, which included scoring two video assessments using test instructions and the opportunity for participants to ask questions. Additionally, Franki et al. [26] found excellent novel vs. expert rater reliability when comparing one expert rater and two non-clinically experienced raters who were familiar with the scale GMFM-88 and who underwent training by scoring 5 videos with the possibility to ask questions [26]. Other clinical tools, such as the Assisting Hand Assessment (AHA) [41,42] or the Pretchl’s General movements and the Hammersmith Neonatal Neurological Examination (HINE) [2,43], require specific and regulated training as an accreditation certificate is mandatory for using these tools. The training of raters seems relevant, as it may influence inter-rater reliability. However, there could be different forms of training that could be used, and investigating which forms of training ensure greater rigor and inter-rater reliability would be very interesting. In the current research, the briefness and format of the training in the use of the TCMS-S may have limited the accuracy of novice raters, although the results do not seem to highlight this.

Furthermore, regarding the procedure of video ratings, the raters in the current study were allowed to pause and review the video as many times as necessary, similar to Franki et al. [26]. In contrast, Kuo et al. [29] and Dewar et al. [44] required videos to be viewed at regular speed and only once in order to simulate in-person assessment. These differences could have influenced why Franki et al. [26] reported excellent inter-rater reliability between novice and expert raters, while Kuo et al. [29] found good agreement. Dewar [44] found excellent inter-rater reliability for the total score of the Balance Evaluation Systems Test (BESTest), but both raters were experts. These findings may indicate that the mode of video viewing influences inter-observer reliability, especially among novice raters [26,27]. In clinical settings where recording material or time for subsequent video analysis are not available [27], a support person may be helpful in securing the patient and scoring the scale. Indeed, the TCMS has been designed to be administered in clinical settings where a careful in-person assessment allows the identification of elements of postural control such as lack of selective control of trunk rotations or difficulty in maintaining balance during a reach. These clinical insights can be useful in setting therapeutic goals or aiding in the selection of a technical aid, but scoring can be challenging during assessment. Therefore, video-recording can be a valuable tool in cases where there is no support person available during the assessment, providing an alternative option for rating [26,45].

This study found that, based on expert inter-rater analysis, the TCMS-S total score had a SEM of 2.0 points and an MDC of 5.7 points (9.8%). These values are consistent with those reported for other versions of the TCMS [12,18,22]. For the total score, Heyrman et al. [18] reported an SEM of 1.68 points and an MDC of 4.66 (8%) points, while Marsico et al. [12] reported an SEM of 1.9 points and an MDC of 5.27 (9%) points, and Ravizzotti et al. [22] reported an SEM of 1.72 and an MDC of 4.83 (8%). Our findings suggest that the MDC between expert raters using the TCMS-S is six points. To ensure independence from measurement error, any changes in the scale score must exceed six points. However, our study did not report on the smallest clinically significant change. Therefore, further longitudinal research is necessary to determine this value [46]. 

Additionally, our results show that novice raters obtained higher SEM (4.2 points) and MDC (11.6 points, 20%) values than expert raters. These findings suggest that experience plays an important role in accurately assessing TCMS-S score. These findings are consistent with results reported for other scales such as the GMFM-88 [26], BESS [29], and the Test Gross Motor Development [27,28]. These results highlight the importance of rater experience in obtaining the most accurate and reliable measurements in clinical trials [26,27,29,44]. Indeed, although agreement among novice raters using the TCMS-S is considered good, the increased SEM and MDC should be considered given the relevance of the assessment in a clinical trial, to assess the functional level of a patient, or to use the data to make clinical decisions. It seems necessary to specify the experience and training of the rater when disseminating the results, as experience conditions the reliability, precision, and sensibility of the ratings [27,29].

Furthermore, the present study revealed that the lowest ICCs for the TCMS-S were observed in the agreement both between novice raters and between novices and experts for the Selective Movement Control subscale (ICC = 0.72 and 0.85, respectively). Moreover, with analyzing TCMS-S total scores and subscales classified by GMFCS levels, it becomes apparent that the Selective Movement Control subscale is the most challenging of the three subscales. Even at GMFCS levels I or II, which obtained the highest scores, the medians do not approach the maximum score. Both the original and German versions of the TCMS have demonstrated similar results, suggesting that this subscale poses a significant challenge for participants, even those with higher functional levels [18,19].

Moreover, this Selective Movement Control subscale has a higher SEM and MDC, even between expert raters. Minor differences, but in the same direction, were noted in previous studies on the original, German, and Tanzanian versions of the TCMS [12,18,22]. These findings could be explained as this subscale is considered the most challenging to assess due to the complexity of the movements involved, such as selective rotations of the upper and lower trunk (item 8 and 9). In addition, the rater must differentiate the quality of the movement, identifying if there are compensations or if the movement is of adequate amplitude. Although all TCMS versions have precise instructions for each item, results suggest that selective movement control subscale requires further attention and even specific training to reach higher consensus when used in clinical or research settings [18,36].

Overall, the TCMS-S has demonstrated excellent reliability among expert evaluators, as well as good reliability among novice raters with minimal training, resulting in a reliable tool for assessing trunk control in the Spanish pediatric population with CP. 

## 5. Conclusions

This study investigated the influence of expertise on the assessment of trunk control in children with CP using the TCMS-S scale. The results showed that regardless of the level of experience, the agreement between raters was good to excellent for the total TCMS-S score and subscales. However, novice raters had slightly lower agreement and higher SEM and MDC than experienced raters. Regarding expert inter-raters scoring, a difference of 6 points in the TCMS-S total score can be considered as a real change in the participant’s trunk control. The Selective Movement Control subscale was found to be the most challenging to assess, even among expert raters. The study highlights the importance of the rater’s experience in achieving the most accurate and reliable measurements and suggests that further training may be necessary to improve the inter-rater reliability of the TCMS-S, particularly for the Selective Movement Control subscale. As a whole, the TCMS-S has demonstrated to be a reliable tool for assessing trunk control in the Spanish pediatric population with CP. 

## Figures and Tables

**Table 1:** Descriptive of participants categorized by diagnosis and disability level based on the Gross Motor Function Classification System (GMFCS).

	GMFCS	Unilateral	Diparesis	Tetraparesis	Ataxic	Total
Age range 6–12	I	10	5	1	0	16
	II	6	12	3	0	21
	III	0	1	5	1	7
	IV	0	0	2	1	3
	TOTAL	16	18	11	2	47
		**Unilateral**	**Diparesis**	**Tetraparesis**	**Ataxic**	**Total**
Age range 12–18	I	13	7	0	0	20
	II	7	6	2	3	18
	III	0	2	4	0	6
	IV	0	0	4	1	5
	TOTAL	20	15	10	4	49

GMFCS, Gross Motor Function Classification System.

**Table 2:** Means and standard deviation obtained by novices and experts of the TCMS were grouped based on the GMFCS level.

TCMS-S	GMFCS I (*n* = 36)		GMFCS II (*n* = 39)		GMFCS III (*n* = 13)		GMFCS IV (*n* = 8)	
	Novice	Expert	Novice	Expert	Novice	Expert	Novice	Expert
Total score (x/58)	45.80 ±4.29	51.73 ±3.96	40.05 ±4.92	43.47 ±6.18	32.92 ±8.82	32.3 ±10.72	20.14 ±9.39	20.64 ±12.5
Static sitting balance (x/20)	18.87 ±1.25	19.16 ±1.17	17.87 ±1.57	17.87 ±2.06	14.84 ±4.57	14 ±4.69	10.71 ±4.32	9.5 ±5.55
Selective movement control (x/28)	17.59 ±3.34	22.86 ±3.18	13.29 ±3.69	16.24 ±4.73	10.92 ±3.83	11.38 ±5.12	4.78 ±3.88	6.21 ±.5.40
Dynamic reaching (x/10)	9.33 ±0.98	9.7 ±0.82	8.88 ±0.89	9.35 ±0.7	7.15 ±1.51	6.92 ±2.26	4.64 ±2.47	4.92 ±3.19

GMFCS, Gross Motor Function Classification System; TCMS-S, Trunk Control measurement Scale Spanish version; Mean ± standard deviation.

**Table 3:** Inter-rater reliability for the Trunk Control Measurement Scale Spanish version: total score and subscales.

EXPERT RATERS					
TMCS-S	ER1	ER2	ICC (95% CI)	SEM	MDC_95_
Total Score	42.1 ± 11.8	44.0 ± 11.9	0.96 (0.88 to 0.98)	2.0	5.7
Static sitting balance	16.7 ± 4.1	17.4 ± 4.2	0.93 (0.87 to 0.96)	1.0	2.7
Selective movement control	16.6 ± 6.7	17.8 ± 7.0	0.95 (0.85 to 0.96)	1.6	4.5
Dynamic reaching	8.8 ± 2.1	8.8 ± 2.0	0.95 (0.92 to 0.96)	0.5	1.3
**NOVICE RATERS**					
**TMCS-S**	**N1**	**N2**	**ICC (95% CI)**	**SEM**	**MDC_95_**
Total Score	40.0 ± 10.6	39.0 ± 9.2	0.82 (0.74 to 0.88)	4.2	11.6
Static sitting balance	16.9 ± 3.7	17.4 ± 3.8	0.78 (0.68 to 0.85)	1.7	4.9
Selective movement control	14.4 ± 6.2	13.3 ± 4.6	0.72 (0.60 to 0.81)	2.8	7.8
Dynamic reaching	8.7 ± 1.9	8.2 ± 2.0	0.74 (0.63 to 0.83)	0.9	2.6
**EXPERT RATERS vs. NOVICE RATERS**					
**TMCS-S**	**Novice 1–2**	**Experts 3–4**	**ICC (95% CI)**	**SEM**	**MDC_95_**
Total Score	39.5 ± 9.5	43.0 ± 11.8	0.93 (0.71 to 0.97)	3.1	8.7
Static sitting balance	17.2 ± 3.5	17.1 ± 4.1	0.96 (0.95 to 0.98)	1.0	2.8
Selective movement control	13.9 ± 5.1	17.2 ± 6.8	0.85 (0.20 to 0.95)	2.3	6.3
Dynamic reaching	8.4 ± 1.8	8.8 ± 2.0	0.93 (0.89 to 0.96)	0.6	1.8

TCMS-S, Trunk Control measurement Scale Spanish version; ER, expert rater; N, novice rater; ICC (95%CI), Intra-class Correlation Coefficient for a Confidence Interval of 95%; SEM, Standard Error of Measurement; MDC, Minimal Detectable Change.

## Data Availability

The data associated with the paper are not publicly available but are available from the corresponding author on reasonable request. For additional information please refer to: javier.lopez3@universidadeuropea.es.
